# Genetic Causes of Oculocutaneous Albinism in Pakistani Population

**DOI:** 10.3390/genes12040492

**Published:** 2021-03-28

**Authors:** Zureesha Sajid, Sairah Yousaf, Yar M. Waryah, Tauqeer A. Mughal, Tasleem Kausar, Mohsin Shahzad, Ali R. Rao, Ansar A. Abbasi, Rehan S. Shaikh, Ali M. Waryah, Saima Riazuddin, Zubair M. Ahmed

**Affiliations:** 1 Department of Otorhinolaryngology Head and Neck Surgery, University of Maryland School of Medicine, Baltimore, MD 21201, USA; zureeshasajid@gmail.com (Z.S.); sairayousaf61@gmail.com (S.Y.); mohsinzoologist@gmail.com (M.S.); sriazuddin@som.umaryland.edu (S.R.); 2 Institute of Molecular Biology and Biotechnology, Bahauddin Zakariya University, Multan 60000, Pakistan; rehansadiq80@bzu.edu.pk; 3 Molecular Biology and Genetics Department, Liaquat University of Medical and Health Sciences, Jamshoro 76090, Pakistan; yarmwaryah@sbbusba.edu.pk (Y.M.W.); aliwaryah@hotmail.com (A.R.R.); aliwaryah@lumhs.edu.pk (A.M.W.); 4 Department of Molecular Biology and Genetics, Shaheed Benazir Bhutto University, Shaheed Benazir Abad 67450, Pakistan; 5 Department of Zoology, Mirpur University of Science and Technology, Mirpur, Azad Jammu and Kashmir 10250, Pakistan; pkmughals2@gmail.com (T.A.M.); ansar.zoology@must.edu.pk (A.A.A.); 6 Department of Zoology, Government Sadiq College Women University, Bahawalpur 63100, Pakistan; tasleemkausar2008@hotmail.com; 7 Department of Biochemistry and Molecular Biology, University of Maryland School of Medicine, Baltimore, MD 21202, USA

**Keywords:** oculocutaneous albinism, OCA, genetic heterogeneity, familial heterogeneity, TYR, OCA2, exome sequencing, Pakistan

## Abstract

Melanin pigment helps protect our body from broad wavelength solar radiation and skin cancer. Among other pigmentation disorders in humans, albinism is reported to manifest in both syndromic and nonsyndromic forms as well as with varying inheritance patterns. Oculocutaneous albinism (OCA), an autosomal recessive nonsyndromic form of albinism, presents as partial to complete loss of melanin in the skin, hair, and iris. OCA has been known to be caused by pathogenic variants in seven different genes, so far, according to all the currently published population studies. However, the detection rate of alleles causing OCA varies from 50% to 90%. One of the significant challenges of uncovering the pathological variant underlying disease etiology is inter- and intra-familial locus heterogeneity. This problem is especially pertinent in highly inbred populations. As examples of such familial locus heterogeneity, we present nine consanguineous Pakistani families with segregating OCA due to variants in one or two different known albinism-associated genes. All of the identified variants are predicted to be pathogenic, which was corroborated by several in silico algorithms and association with diverse clinical phenotypes. We report an individual affected with OCA carries heterozygous, likely pathogenic variants in *TYR* and *OCA2*, raising the question of a possible digenic inheritance. Altogether, our study highlights the significance of exome sequencing for the complete genetic diagnosis of inbred families and provides the ramifications of potential genetic interaction and digenic inheritance of variants in the *TYR* and *OCA2* genes.

## 1. Introduction

Melanosomes are the cellular organelles (~500 nm in diameter) that are involved in the synthesis, storage, and transportation of melanin pigment in various tissues. This includes but is not limited to the skin, retinal pigment epithelium cells (RPE), and stria vascularis of the inner ear in mammals [1]. The multi-step melanocyte development process is comprised of fate specification, migration, and differentiation in a highly controlled temporospatial manner [2,3]. Melanocytes operate under the control of multiple gene regulatory networks for the sake of optimal functionality [4]. Significant aberrations at any stage of melanocyte, melanosome, or melanin synthesis and their inter- and intracellular transport can lead to heterogeneous pigmentation disorders in humans. Insufficient or lack of pigmentation makes the affected individuals more vulnerable to ultraviolet-mediated skin abrasions and prone to developing life-threatening conditions, e.g., melanoma and skin carcinoma [5,6]. Oculocutaneous albinism (OCA) is a pigmentation disorder that presents a lack of pigment in the skin, eyes, and hair follicles [7]. Worldwide, albinism affects approximately every 1 in 17,000 individuals, though the prevalence of OCA subtypes varies among different populations [8,9]. Additionally, in humans, OCA can manifest as part of a multi-organ syndrome or an isolated (non-syndromic; nsOCA) clinical entity. Clinical features of OCA include nystagmus, photophobia, strabismus, foveal hypoplasia, visual deficits, and misrouting of the optic nerve at the chiasm [10].

Among the known genetic causes of nsOCA, variants in *TYR* and *OCA2* are the most prevalent worldwide [11,12,13]. *TYR* encodes a transmembrane glycoprotein tyrosinase that resides in the melanosome membrane and plays a vital role in catalyzing the initial and rate-limiting steps of melanin synthesis [14]. In contrast, the *OCA2*-encoded transmembrane protein is involved in the maintenance of melanosome pH and activity of the chloride-ion channels [15,16]. In recent years, advances in massively parallel sequencing approaches have expedited the process of gaining insight into the genetic basis of Mendelian disorders. These massive genetic profiling projects have also brought to light the significance and severity of several crucial issues. These issues include the variability in disease onset and progression rate, incomplete penetrance, and high inter- and intra-familial genetic heterogeneity for Mendelian disorders, including OCA [17,18]. Recently, a rhesus macaque model of albinism revealed biallelic variants in both *TYR* and *OCA2* that have been used to carry out foveal development studies and preclinical trials of new therapies for OCA [19]. 

The inheritance of pathogenic variants at different loci, which triggers the disease commencement [20], could also be suggestive of some level of genetic association or functional corroboration between these loci [21]. The current study strives to find the single, double, or multiple disease-associated variants in known *OCA* genes in inbred Pakistani families with diverse ethnicities with the goals of providing molecular diagnosis and identifying potential genetic interactions between known *OCA* genes in humans.

## 2. Material and Methods

### 2.1. Ethics Statement

After receiving study approval by the Institutional Review Boards and Ethics committees (HP-00061036, approved on 20 January 2020) at participating institutes (Universities of Maryland, Baltimore, MD, USA, Liaquat University of Medical and Health Sciences, Jamshoro, Bahauddin Zakariya University, Multan, and Mirpur University of Science and Technology, Mirpur, Azad Jammu and Kashmir, Pakistan), families that were segregating OCA were identified and ascertained from the Sindh, Kashmir, and Punjab provinces of Pakistan. All the protocols used to carry out this study ensued the Declaration of Helsinki. Written informed consent was also obtained from all participants before enrollment. Peripheral venous blood samples were collected from all the participating individuals for the genomic DNA extraction.

### 2.2. Clinical Examination 

We recorded a detailed clinical history by interviewing subjects at the time of enrollment. Photographs were taken to document the pigmentation phenotype of the skin, eyes, and hair. Ophthalmic evaluations consisting of a visual acuity test, slit lamp microscopy, fundoscopy, and optical coherence tomography were performed on the available subjects by clinicians.

### 2.3. Sanger Sequencing of Known OCA Genes

For the genetic screening, we amplified both the coding and exon-intron junction regions of all the exons of known nonsyndromic *OCA* genes through PCR using Econotaq DNA Polymerase (Bioresearch Technologies, Radnor, PA, USA). The samples were then subjected to Sanger sequencing as previously described [22]. Allele-specific PCR was also used to confirm results for a few variants [23]. 

### 2.4. Bioinformatic Analysis

We used Varsome [24] for classification of the identified variants in accordance with the American College of Medical Genetics and Genomics (ACMG) guidelines [25]. We also used several other in silico algorithms, including DANN (which presents a score based upon deep neural networks) [26], REVEL (which predicts pathogenicity using 13 independent programs: MutPred, FATHMM v2.3, VEST v3.0, Polyphen-2, SIFT, PROVEAN, MutationAssessor, MutationTaster, LRT, GERP++, SiPhy, phyloP, and phastCons) [27], MetaSVM (which shows the combinatory result of nine pathogenicity prediction programs and 1KG allele frequency database) [28], and DEOGEN2 (which integrates information related to amino acid, protein structure, domain function, and molecular pathway) [29] to evaluate the impact of identified variants on the encoded proteins. Finally, Clustal Omega was used to show protein conservation across several species, and protein 3D structures were generated and visualized by Phyre2 and Chimera, respectively.

## 3. Results

### 3.1. Clinical Manifestation

**Figure 1:**
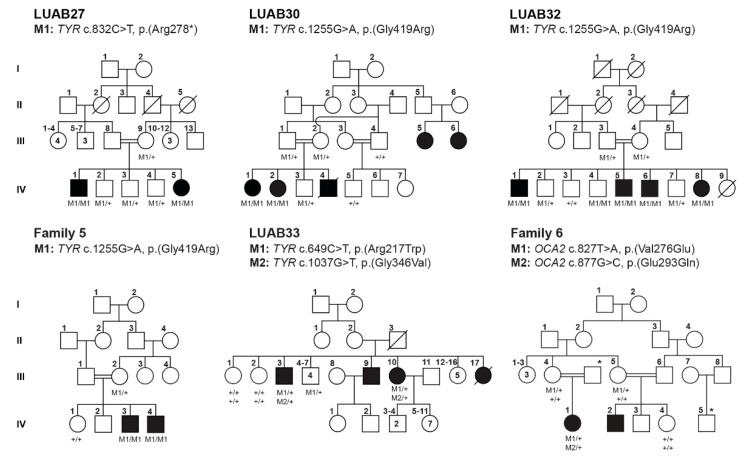
Pedigrees of Pakistani families segregating nonsyndromic OCA due to single-gene variants are shown. Genotypes are mentioned below each sequenced individual, while the identified variants and gene names are listed on top of each pedigree. Empty and filled symbols represent the normal and affected individuals, respectively. A double line between two individuals represents a consanguineous marriage.

**Figure 2:**
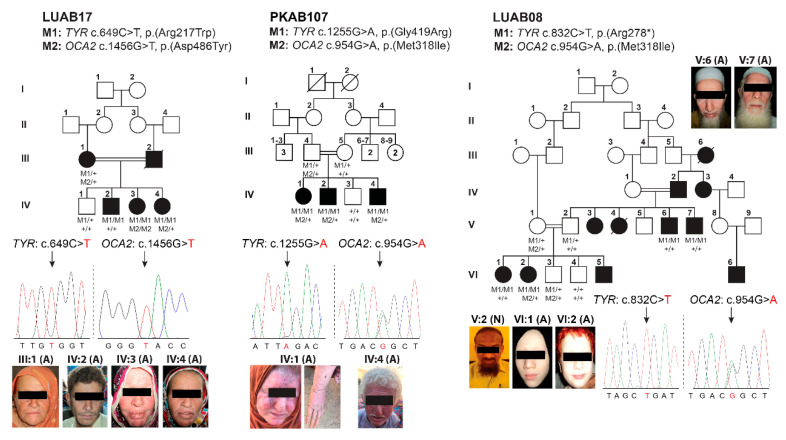
Family pedigrees of Pakistani OCA families with multi-gene variants are shown. Genotypes are mentioned below each sequenced individual, while the identified variants and gene names are listed on top of each pedigree. Empty and filled symbols represent the normal and affected individuals, respectively. A double line between two individuals represents a consanguineous marriage. Sequenced chromatograms of the affected individuals along with photographs are displayed below pedigrees with the cDNA change mentioned in red color.

**Figure 3:**
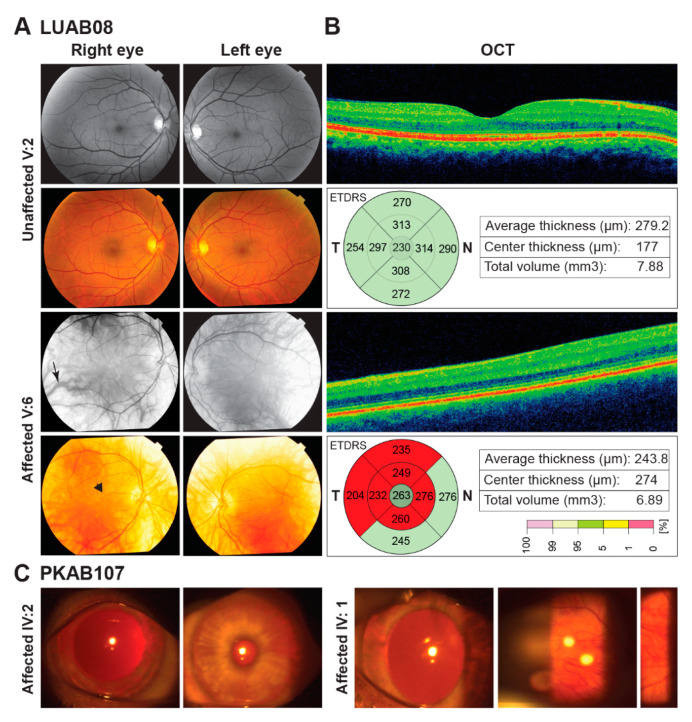
Fundus photographs and optical coherence tomography (OCT) images of OCA affected individuals. **A.** Fundus photographs of normal (V:2) and affected (V:6) individual of family LUAB08. Affected (V:6) represents albinotic fundus, prominent choroidal vasculature (arrow), foveal hypoplasia (arrowhead), and thin retinal thickness. **B.** OCT images of normal (V:2) and affected (V:6) individual of family LUAB08. **C.** Slit lamp microscopy in the affected individuals (IV:1 and IV:2) of family PKAB107 represent iris transillumination and albinotic fundus.

We enrolled nine consanguineous families segregating OCA (Figure 1 and Figure 2) from different regions of Pakistan, including the Sindh, Kashmir, and Punjab provinces. Affected individuals from all of the recruited families presented with cardinal features of OCA symptoms that included hypopigmentation of the skin, white to yellow-white hair color, lightly pigmented eyes, reduced vision, iris transillumination, nystagmus, and photophobia (Table 1). Representative fundus and optical coherence tomography (OCT) images of the affected (V:6) and unaffected individuals of family LUAB08 are shown in Figure 3. As can be seen in contrast to the well-developed fovea/macula with normal pigmentation in the unaffected individual (V:2; aged 45 years), the fundus images of the affected individual (V:6, aged 47 years) show foveal hypoplasia (arrowhead) with prominent choroidal vasculature (arrow) and variable levels of pale-pigmented retinal epithelial layer (particularly outside the vascular arcs) (Figure 3A). Similarly, OCT of the unaffected individual (V:2) show a normally structured fovea, foveal pit, and all retinal layers (Figure 3B). Conversely, the OCT image of the affected individual (V:6) revealed a lack of outer nuclear layer widening at the fovea and an absence of the foveal pit (Figure 3B). Furthermore, the mean of the macular thickness (shown by the macular thickness map using 1, 3, and 6 mm ETDRS circles describing inner fovea, inner, and outer macula, respectively), was reduced in the affected individual (V:6) as compared to unaffected sibling (Figure 3B). Slit lamp microscopy in the affected individuals (IV:1 and IV:2) of family PKAB107 showed iris transillumination and albinotic fundus (Figure 3C) that is consistent with the albinism phenotype.

Intriguingly, the affected individual, III:1, of family LUAB17 is heterozygous for both *TYR* and *OCA2* variants. This raises the question of digenic inheritance of the OCA phenotype and genetic interaction between these two known *OCA* genes.

### 3.2. Identification of Pathogenic Variants in OCA-Affected Families

Next, to determine the genetic causes of OCA segregating in these nine families, Sanger sequencing of coding and non-coding exons of all six known *OCA* genes (*TYR* (*OCA1*), *OCA2*(*OCA2*), *TYRP1* (*OCA3*), *SLC45A2* (*OCA4*), *SLC24A5* (*OCA6*) and *C10ORF11* (*OCA7*)) was performed for the proband of each family. Both homozygous and compound heterozygous variants were identified in these genes. All genes with a minor allele frequency of <0.001 in the gnomAD database were considered for segregation analysis in all the participating family members. Using this approach, we were able to resolve the locus heterogeneity in all families. In five families, the variants in *TYR* were associated with the disease phenotype, while compound heterozygous variants in *OCA2* are responsible for OCA in one family (Figure 1; Table 2). Four previously reported variants, c.832C > T (p.(Arg278*)), c.1255G > A (p.(Gly419Arg)), c.649C > T (p.(Arg217Trp)), and c.1037G > T (p.(Gly346Val)) in *TYR* were found segregating with the OCA phenotype in the homozygous or compound heterozygous (family LUAB33) state in five families (Figure 1). Two novel variants, c.827T > A (p.(Val276Glu)), and c.877G > C (p.(Glu293Gln)), of *OCA2* were found in family 6 (Figure 1).

Intriguingly, in family LUAB17, the affected individuals harbor rare variants both in *TYR* and *OCA2* genes that are predicted to be deleterious, while the unaffected family member is a carrier of the identified *TYR* variant (Figure 2; Table 1). In family LUAB17, we identified the segregation of two previously reported pathogenic missense variants (c.649C>T: p.(Arg217Trp); c.1456G>T: p.(Asp486Tyr)) of *TYR* and *OCA2,* respectively, in multiple genotype states (Figure 2). Importantly, the affected individual III:1 (white skin and hair and brown iris color) was found to be heterozygous for both TYR and OCA2 variants, and thus poses the question of digenic inheritance of an OCA phenotype and genetic interaction between these two known OCA genes. Although screening of both coding and exon-intron splicing regions did not reveal any additional pathogenic variant either in *TYR* or *OCA2* in all the affected individuals of family LUAB17, we cannot rule out the possibility of deep intronic variants of either gene acting *in trans* with the identified variants. 

**Figure 4:**
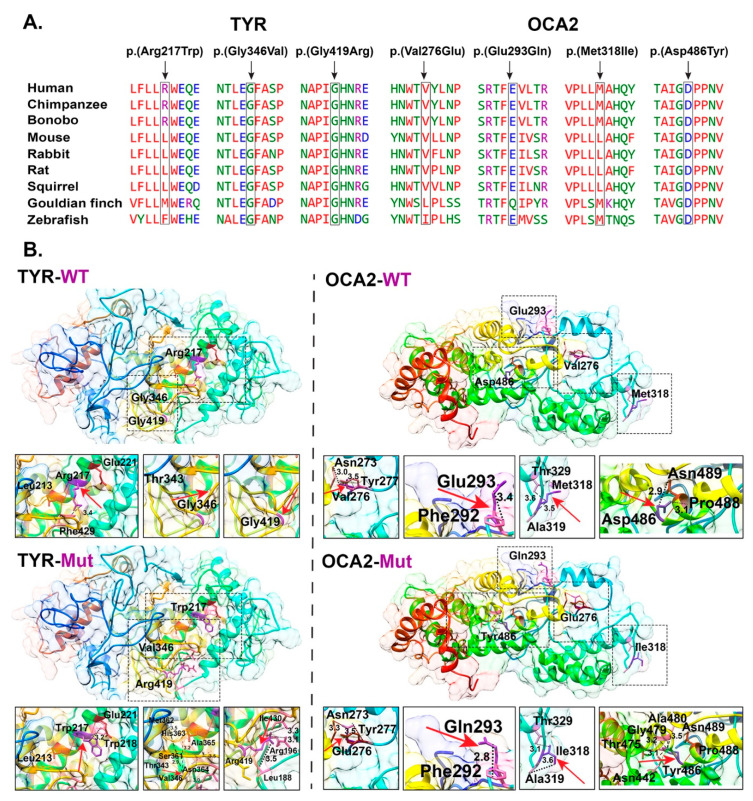
Clustal alignment and 3D protein modeling of TYR and OCA2 variants. **A.** Clustal alignment of TYR and OCA2 sequence across a number of species. **B.** Pair-wise comparison of TYR and OCA2 wild type (top) and mutant (bottom) residues (marked with arrows), predicted changes. Protein secondary structure is shown in ribbon and hydrophobic surface representation. Residues of interest are shown in purple, and hydrogen bonds are shown as solid red lines. Dotted black lines are used to show the distance of residue of interest with nearby residues. TYR: NM_000372.5 and OCA2: NM_000275.3.

Finally, all the affected individuals of families PKAB107 and LUAB08 were found to be homozygous for the known variants (c.1255G>A: p.(Gly419Arg); c.832C>T (p.(Arg278*)) of *TYR*, respectively (Figure 2). Some of the participating members of these families were also heterozygous for a rare variant (c.954G>A; p.(Met318Ile)) of *OCA2* (Figure 2). Although the p.(Met318Ile) does not have an evolutionarily conserved residue (Figure 4A), it has high Combined Annotation Dependent Depletion (CADD) scores and was predicted pathogenic by few *in silico* algorithms (Table 2). However, the individual III:4 of family PKAB107 and individuals V:1; VI:3 of family LUAB08 that are heterozygous for *TYR,* and the p.(Met318Ile) *OCA2* (Figure 2), have no pigmentation problems. On the other hand, in the exome data of 141,334 individuals listed in the gnomAD database, only one homozygote (minor allele frequency: 4.28 × 10^−4^) was found. With the current evidence that lacks a detailed pigmentation phenotype and takes into account the description of the p.(Met318Ile) homozygote without functional studies, we cannot conclude if p.(Met318Ile) would be pathogenic or not in the homozygous state. However, we included this rare variant in our in silico 3-dimensional molecular modeling to assess the potential impact on the OCA2 protein along with other identified variants (Figure 4). 

### 3.3. Protein Modeling of TYR and OCA2 Variants 

Collectively, we have identified six potential missense variants of *TYR* and *OCA2* in our OCA families (Table 2). All these variants are either absent or have very low frequencies in the gnomAD database, are predicted by several in silico algorithms to be damaging (Table 2), and also most of them have high conservation across multiple species (Figure 4A). To assess the predicted impact of the identified OCA1-associated variants on the encoded tyrosinase enzyme secondary structures, we performed 3D molecular modeling with Phyre-2 software. 

The p.(Arg217Trp) missense variant of TYR is predicted to be present in the copper-binding domain, which is vital for the oxidoreductase activity of the encoded tyrosinase enzyme. The WT arginine residue at position 217 is located on the protein surface and is predicted to form hydrogen bonds with p.Glu221 and p.Leu213 residues as well as a salt bridge with p.Glu221. Due to differences in the structure and properties, the p.(Arg217Trp) replacement is predicted to induce a loss of ionic interactions with other residues (Figure 4B). Similarly, the p.(Gly346Val) variant found in family LUAB33, is also located within the tyrosinase copper-binding domain. Replacement of glycine at position 346 with valine is predicted to introduce new hydrogen bonds and force the local protein backbone into an improper conformation (due to size and charge differences between the WT and mutated residues) (Figure 4B). Finally, the glycine residue at position 419 is buried in the lumenal melanosome residues of the repeat stretch of the tyrosinase enzyme. Replacement of the glycine with a bigger and positively charged arginine residue at position 419 is predicted to disrupt the protein folding and secondary structure as well as introduce aberrant ionic interactions (Figure 4B).

We also modeled the OCA2-associated missense variants. The p.Val276Glu variant of OCA2 is predicted to replace the neutral (valine) residue with a negatively charged (glutamic acid) residue, which may cause repulsion of ligands or other residues of the same charge. Furthermore, differences in the size and hydrophobicity of valine and glutamic acid are also predicted to result in the loss of hydrophobic interactions (Figure 4B). Similarly, the p.Glu293Gln variant is predicted to result in a loss of charge and associated interactions with other residues in the core of the encoded protein (Figure 4B). The p.Met318Ile variant of uncertain significance found in families, PKAB107 and LUAB08 (Figure 2), is located in the alpha-helix loop. Replacing the residue isoleucine (p.Met318Ile) would cause the protein to resist the alpha-helices secondary structure and would likely cause spacing in the secondary structure due to the small size (Figure 4B). Finally, the p.Asp486Tyr change is predicted to negatively impact the protein folding and ionic interactions due to the differences in size and charge among amino acids (Figure 4B). 

## 4. Discussion

OCA is a clinically and genetically heterogeneous disorder that segregates in an autosomal recessive pattern in humans. Unlike any other genetic disorders caused by single-gene pathogenic variants (e.g., cystic fibrosis), non-syndromic presentations of OCA are already linked with eight distinct autosomal genetic loci. Among these genetic links, variants in the *TYR* (OCA1) and *OCA2* (OCA2) genes account for a majority of the OCA cases worldwide, including those in Pakistan [22,23]. Besides the hundreds of homozygous variants, there are many paragons illustrating the inheritance of the compound heterozygous variants of *TYR* and *OCA2* and their linkage to the OCA phenotype. Further, challenges in uncovering the pathological variant underlying disease etiology are imposed by the inter- and intra-familial locus heterogeneity. Our study illustrates nine examples of familial locus heterogeneity for nonsyndromic OCA. We describe four OCA families (LUAB27, LUAB30, LUAB32, and Family 5) that harbor two known *TYR* variants (p.(Arg278*); p.(Gly419Arg)) that segregate in the homozygous state. The affected individuals of family LUAB33 inherited two heterozygous variants (p.(Arg217Trp); p.(Gly346Val)) of *TYR* in trans configuration from their parents. Similarly, two novel compound heterozygous variants (p.(Val276Glu); p.(Glu293Gln)) of OCA2 were found in the affected individuals of Family 6. 

Besides these cases of single-gene variants, we also found variants of both *TYR* and *OCA2* in different zygosity combinations within family LUAB17. The obtained combination of OCA variants might interact in a novel manner to generate the observed OCA phenotypes (e.g., individual III:1 family LUAB17), accentuating the significance of genetic interactions towards OCA etiology. Possible digenic inheritance of OCA variants has also previously been proposed in other populations. For instance, five cases of OCA harboring distinct allelic combinations of *TYR*, *OCA2*, and *SLC45A2* have been reported in the Chinese population [18]. This only serves to emphasize the importance of considering the implications of genetic interaction between multiple known *OCA* genes during embryonic development. Although digenic or oligogenic inheritance has not been proven for albinism, it has been reported for other Mendelian disorders, e.g., familial microscopic hematuria [32] and digenic familial exudative vitreoretinopathy [33]. For example, Bardet–Biedl syndrome is a well-studied vision disorder with oligogenic inheritance, genetic interactions, and phenotype modifications [34,35]. Currently, the sample size of OCA cases with oligogenic variants is not large enough for a meaningful evaluation of phenotype modifications. However, our study contributes useful genetic information towards such an endeavor.

## 5. Conclusions

In conclusion, our study expands the genetic spectrum of OCA in the Pakistani population, aids in the complete genetic testing and counseling of families inheriting variants of OCA genes, and raises the question of whether a potential genetic interaction and digenic inheritance of variants in *TYR* and *OCA2* genes can exist. 

## Figures and Tables

**Table 1:** Clinical findings of OCA-affected families from Pakistan.

Family	Subject ID	Sex	Age (Years)	Status	Ethnicity	Hair Color	Skin Tone	Iris Color	Visual Acuity		Refractive Error	Photophobia	Nystagmus	Fundus	Foveal Hypoplasia
									Right	Left					
**LUAB27**	IV:2	M	16	Normal	Sindh	Black	Wheat	Black	6/6	6/6	Normal	No	No	Normal	Formed
	IV:1	M	19	Affected		White	Pinkish white	Brown	6/40	6/40	Hypermetropia	Yes	Yes	Pigmented	Poorly formed
	IV:5	F	8			White	White	Brown	6/20	6/20	Hypermetropia	Yes	Yes	Pigmented	Poorly formed
**LUAB30**	IV:3	M	4	Normal	Sindh	Black	Wheat	Black	NA	NA	NA	No	No	NA	NA
	IV:1	F	5	Affected		White	White	Brown	NA	NA	NA	Yes	Yes	NA	NA
**LUAB32**	IV:3	M	24	Normal	Sindh	Black	Wheat	Brown	6/6	6/6	Normal	No	No	Normal	Normal
	IV:1	M	35	Affected		White	Pinkish white	Gray blue	C.F	C.F	Hypermetropia	Yes	Yes	Small pigmented	Poorly formed
	IV:5	M	26			White	Pinkish white	Gray blue	6/60	6/60	Hypermetropia	Yes	Yes	NA	NA
	IV:6	M	23			White	Pinkish white	Gray blue	6/20	6/40	Hypermetropia	Yes	Yes	Pigmented	Absent
	IV:8	F	28			White	Pinkish white	Gray blue	NA	NA	NA	Yes	Yes	NA	NA
**Family 5**	IV:1	F	17	Normal	Kashmir	Black	White	Brown	NA	NA	NA	No	No	Normal	No
	IV:3	M	23	Affected		White	White	Blue	NA	NA	NA	Yes	Yes	NA	NA
	IV:4	M	26			White	White	Blue	NA	NA	NA	Yes	Yes	NA	NA
**Family 6**	III:4	F	40	Normal	Kashmir	Black	Brown	Brown	NA	NA	NA	No	No	Normal	No
	IV:1	F	5	Affected		White	White	Gray blue	NA	NA	NA	Yes	Yes	NA	NA
**LUAB08**	V:2	M	45	Normal	Sindh	Black	Wheat	Brown	6/6	6/7	Normal	No	No	Normal	Formed
	V:6	M	47	Affected		White	White	Gray blue	C.F	C.F	Hypermetropia	Yes	Yes	Pigmented	Poorly formed
	V:7	M	41			White	White	Gray blue	4/60	4/60	Hypermetropia	Yes	Yes	Albinotic	Yes
	VI:1	F	26			White	Pinkish white	Brown	C.F	C.F	Hypermetropia	Yes	Yes	Pigmented	Absent
	VI:2	F	24			White	White	Brown	C.F	C.F	Hypermetropia	Yes	Yes	Pigmented	Absent
**LUAB17**	III:1	F	68	Affected	Sindh	White	White	Brown	6/60	6/40	Hypermetropia	Yes	Yes	NA	NA
	IV:2	M	31	Affected		Brown	Pale white	Brown	6/20	6/20	Hypermetropia	Yes	Yes	NA	NA
	IV:3	F	36			Yellow white	Pinkish white	Gray	6/10	6/10	Hypermetropia	Yes	Yes	NA	NA
	IV:4	F	46			Brown	Pale white	Gray blue	NA		NA	Yes	Yes	NA	NA
**PKAB107**	IV:1	F	25	Affected	Punjab	White	Pinkish white	Gray blue	6/60	6/60	Hyperopic	Yes	Yes	Albinotic	Yes
	IV:2	M	30	Affected		White	Pinkish white	Gray blue	NA	NA	NA	Yes	Yes	Albinotic	Yes

C.F: Counting finger.

**Table 2:** List of genetic variants found in Pakistani OCA families.

Family	Gene	cDNA Change	AA Change	gnomAD	CADD	DANN	REVEL	MetaSVM	DEOGEN2	ACMG Classification	Reference
LUAB27	*TYR*	c.832C > T	p.(Arg278*)	0.000169	39	0.99	NA	NA	NA	Pathogenic (PM2, PVS1, PP3, PP5)	[18]
LUAB30		c.1255G > A	p.(Gly419Arg)	0.000060	29	0.99	Pathogenic	Damaging	Damaging	Pathogenic (PS1, PM1, PM2, PP2, PP3, PP5)	[30]
LUAB32											
Family 5											
LUAB33		c.649C > T	p.(Arg217Trp)	0.000191	23	0.99	Benign	Damaging	Damaging	Pathogenic (PM1, PM2, PM5, PP2, PP5, BP4)	[23]
		c.1037G > T	p.(Gly346Val)	Not found	33	0.99	Pathogenic	Damaging	Damaging	Pathogenic (PM2, PM5, PP2, PP3, PP5)	[31]
Family 6	*OCA2*	c.827T > A	p.(Val276Glu)	Not found	19	0.97	Benign	Damaging	Damaging	Uncertain significance (PM2, PP2, PP3)	This study
		c.877G > C	p.(Glu293Gln)	Not found	22	0.98	Benign	Damaging	Tolerated	Uncertain significance (PM2, PP2, PP3)	This study
LUAB08	*TYR*	c.832C > T	p.(Arg278*)	0.000169	39	0.99	NA	NA	NA	Pathogenic (PM2, PVS1, PP3, PP5)	[18]
	*OCA2*	c.954G > A	p.(Met318Ile)	0.000428	22	0.98	Benign	Damaging	Tolerated	Uncertain significance (PP2, PM2)	This study
LUAB17	*TYR*	c.649C > T	p.(Arg217Trp)	0.000191	23	0.99	Benign	Damaging	Damaging	Pathogenic (PM1, PM2, PM5, PP2, PP5, BP4)	[23]
	*OCA2*	c.1456G > T	p.(Asp486Tyr)	0.000023	30	0.99	Pathogenic	Damaging	Damaging	Uncertain significancePM2, PP2, PP3	[11]
PKAB107	*TYR*	c.1255G > A	p.(Gly419Arg)	0.000060	29	0.99	Pathogenic	Damaging	Damaging	Pathogenic (PS1, PM1, PM2, PP2, PP3, PP5)	[30]
	*OCA2*	c.954G > A	p.(Met318Ile)	0.000428	22	0.98	Benign	Damaging	Tolerated	Uncertain significance (PP2, PM2)	This study

**PVS1:** Pathogenic Very Strong, null variant (nonsense, frameshift, canonical ±1 or 2 splice sites, initiation codon, single or multiexon deletion) in a gene where loss-of-function (LOF) is a known mechanism of disease. **PS1:** Pathogenic Strong, the same amino acid change as a previously established pathogenic variant regardless of nucleotide change. **PM1:** Pathogenic Moderate, located in a mutational hot spot or critical and well-established functional domain (e.g., the active site of an enzyme) without benign variation. **PM2:** Pathogenic Moderate, absent from controls (or at extremely low frequency if recessive) in Exome Sequencing Project, 1000 Genomes Project, or Exome Aggregation Consortium. **PM5:** Pathogenic Moderate, novel missense change at an amino acid residue where a different missense change determined to be pathogenic has been seen before. **PP2:** Pathogenic Supporting, missense variant in a gene that has a low rate of benign missense variation and in which missense variants are a common mechanism of disease. **PP3:** Pathogenic Supporting, multiple lines of computational evidence support a deleterious effect on the gene or gene product (conservation, evolutionary, splicing impact, etc.). **PP5:** Pathogenic Supporting, a reputable source recently reported variant as pathogenic, but the evidence is not available to the laboratory to perform an independent evaluation. **BP4:** Benign Supporting, multiple lines of computational evidence suggest no impact on gene or gene product (conservation, evolutionary, splicing impact, etc.). **NA:** Not Available.
